# The Poor in London

**Published:** 1894-01-20

**Authors:** 


### THE POOR IN LONDON.

In February, 1893, Sir Stuart Knill, then Lord Mayor, summoned a conference, at the Mansion House, of the titular heads of the churches and religious communities in London and various representative laymen, to consider how far it was possible to secure the following objects:—(1) The union of all religious organisations in each district for the purpose of grappling with the poverty by systematic visitation, and avoiding overlapping, and (2) the formation of an inter-denominational representation committee in each district, with the members of Parliament and the London County Councillors, or some other representative laymen, as chairman and vice-chairman respectively, to organise and direct the work in the manner best adapted for the district. A committee was appointed to discuss these questions, and after many deliberations and much correspondence, it has recently formulated a report, which embodies the following resolutions: "That this conference, representing various religious communities, is of opinion that the best way to discover and meet the sufferings of the poor in London is by concerted action of all the religious and benevolent agencies in the several parishes, and, therefore, suggests that some such mode of co-operation as has worked successfully in North Kensington, under the name of 'The Friendly Workers Among the Poor,' should be formed as opportunity may serve in the different districts of London by central committees representative of all denominations." The committee further reported, as the result of their inquiries, that it was possible in many districts of the metropolis to secure the co-operation of all the religious and charitable agencies for the relief of poverty in the metropolis. The Bishop of London recently presided over a meeting of the original conference of titular heads representing the Churches of all denominations, at which it was determined to empower the committee to take steps, in conjunction with all the religious and benevolent agencies in four other areas favourable to the present attempt, with a view to the establishment of the necessary machinery for carrying the Friendly Workers' scheme into operation at once. The committee were impressed by the feeling that the real difficulty in London arises from the constant competition of the different religious and benevolent bodies in trying to meet the distress that exists around them. They fully recognised the perfect good feeling with which all these bodies are working, but they say, "They cannot shut their eyes to the fact that the gross result of their work is that, although it may relieve the poverty, it also has a tendency to create the poverty, inasmuch as it opens indefinite possibilities of procuring aid from different religious persons and sections of the community." The report goes on to say: "The difficulty of united action arises very largely from the almost complete separation existing between the Church of England and other religious bodies. Of course, every religious community regards philanthropy as a paramount duty, and would not be willing to deprive itself of the privilege of relieving its own poor; whilst the Church of England, from its parochial organisation, considers itself, and is considered, to have the care, not only of members of the Church of England, but of all parishioners who are in circumstances to need help. The merit of the plan proposed by the committee is that, where it is adopted, there will be free intercourse and communication between all persons distributing relief, so that there will be no possibility of competition being created between the two, and exciting in the recipients of relief the very material desire to get the most out of both. The very smallest rivulet of help that is at present being poured into the several districts must be co-ordinated and brought into line with the general work, if the present evils affecting the poor in London are to be prevented. Inter-denominational committees in every district consequently present the most hopeful prospect of solving the problem. An additional merit of the plan proposed is that it supplements the knowledge of the parochial clergy and others and of the various organisations which deal with the diverse cases which arise. For nearly every wrong in the social sphere there exists a remedy in London, if it can only be found, and the creation of inter-denominational committees would put all in a position to find out readily where the remedy lies. The work of the inter-denominational committees ought to be much supplemented by the services of a large number of men and women who have spare time and a desire to do good. Heretofore it has been difficult or impossible to bring the leisured classes sufficiently in contact with the poor; but the committee believe that, as the plan extends, very many such people will be attracted to the work, and so much good may result, not only to the poor, but those above them." With the view of facilitating the work of the committee, the Lord Mayor at the Mansion House will welcome offers of co-operation from all who may be willing to take part in the work as chairmen or secretaries of district Friendly Workers' committees, or from clergymen and laymen who may be desirous of securing the selection of a particular district as one of the four areas to which the inter-denominational system is shortly to be applied by the committee appointed by the heads of Churches of all denominations.

